# Immunization with recombinant rHc23 partially protects lambs against trickle infections by *Haemonchus contortus*

**DOI:** 10.1186/s12917-019-2084-5

**Published:** 2019-09-18

**Authors:** María Elena González-Sánchez, Melissa Ndombasi-Bokuy, Montserrat Cuquerella, José María Alunda

**Affiliations:** 1 Department of Animal Health, Faculty of Veterinary Medicine, Universidad Complutense, Avda. Puerta de Hierro s/n, 28040 Madrid, Spain; 2 Department of Statistics and Operational Research III, Faculty of Statistical Studies, Universidad Complutense, 28040 Madrid, Spain

**Keywords:** Haemonchosis, Lambs, Vaccination, Recombinant, rHc23, Immunomodulator, LPS

## Abstract

**Background:**

Haemonchosis is one of the most economically important parasitic diseases affecting small ruminants all over the world. Chemotherapeutic control has several shortcomings (limited anthelmintic arsenal, frequent resistance) and is hardly affordable by many farm economies. A recombinant antigen (rHc23) was shown to induce significant protection in vaccination trials with single dose challenges and different adjuvants.

**Results:**

Lambs were vaccinated with 100 μg rHc23/dose + bacterial immunostimulant (BI) (LPS from *Escherichia coli* + *Propionibacterium acnes* extract) (days − 2, 0, 7 and 14) and subjected to a trickle infection with two dosages [6x, 1000 infective larvae (L3) or 6x, 2000 L3]. Vaccinated lambs showed a significant antibody response against rHc23 and *Haemonchus contortus* soluble extract as assessed by ELISA and Western blot (WB). Fecal egg counts (epg) along the experiment of vaccinated and BI treated lambs were significantly reduced. All vaccinated animals showed total egg output and abomasal helminth burdens (median, average) lower than those from unvaccinated or BI-treated animals lambs although differences were not statistically significant.

**Conclusions:**

Vaccination with 100 μg rHc23/dose + BI against *H.contortus* trickle infections apparently induced lower epg values and helminth burdens at the end of the experiment. Intragroup individual variations did not allow to obtain conclusive results and more research is needed including adjuvants and larger groups of animals to validate the potential value of rHc23 as candidate to develop a recombinant vaccine for lambs haemonchosis.

## Background

Blood-sucking abomasal *Haemonchus contortus* is probably the most economically important nematode affecting small ruminants [1] and it is considered responsible of the 15% of all gastrointestinal diseases of sheep (http://www.fao.org). Infections in ewes cause weight loss, decreased fertility and lower wool growth and milk production. Lambs are more severely affected and infections are frequently fatal. Control relies mainly on the use of anthelmintics. However, chemotherapeutic arsenal is quite reduced and resistance to all known antiparasitic groups has been reported [2]. Slimmed farm economies, social concerns on the presence of residues in the environment and safety of animal products for human consumption require the establishment of sustainable livestock production. Moreover, expected returns do not favor investments in new anthelmintics. Under natural conditions lambs are able to develop a partial resistance to challenge after repeated exposition to *H.contortus* infections and significant levels of protection have been achieved in a number of trials with different isolated antigens both hidden and exposed [3–5] although the success with their recombinant counterparts has been modest [4, 6]. Immunization of 5–6 months old lambs with the rHc23 recombinant antigen elicited significant protection (> 70% in abomasal helmith burden and > 80% in fecal egg out) against challenge [7] particularly with aluminum hydroxide adjuvant [8]. Most experiments exploring the immunoprophylaxis of haemonchosis have been carried out using single dose challenge. Under natural conditions weaned lambs are exposed to repeated infections during grazing. These repeated exposures to the helminth could interfer with the protective sheep immune response thus leading to uncontrolled infections. With the aim of refining vaccination against lamb haemonchosis with rHc23, a preliminary test of the potential immunoprophylactic value of the recombinant against trickle infections with *H.contortus* has been performed. Present manuscript presents the results obtained when challenging vaccinated (rHc23+ BI) lambs with 1000 L_3_ or 2000 *H.contortus* administered 3 times/week, 2 consecutive weeks.

## Results

**Fig. 1:**
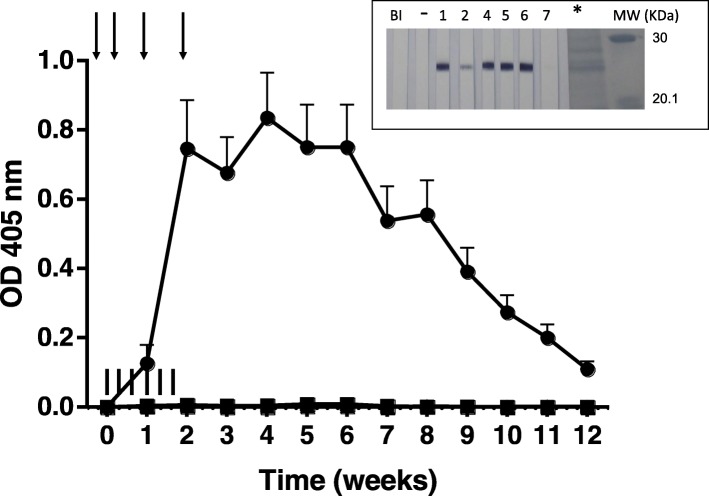
Serum specific IgG response of experimental lambs subjected to 1000 L3/ dose trickle *Haemonchus* infection against rHc23 along the experiment. Group 1 (●), Group 2 (■) and Group 3 (○). Data given (Optical density, OD) are means ± standard error of the mean (SEM) of the individual OD values from each animal and time point (Group 1: 6 lambs; Group 2: 7 lambs; Group 3: 4 lambs). Insert: Immune recognition of ASE of *H.contortus*. Lanes 1–7: individual sera of vaccinated lambs (Group 1); BI: pooled sera from adjuvant control group (Group 2); (−): pooled sera of lambs from untreated + infected group (Group 3). Arrows: days of vaccination. Vertical bars: days of infection. MW: molecular weight markers. *: Membrane stained with Amido black

Entrefino lambs were vaccinated with four doses of 100 μg rHc23 + BI and challenged with a *H.contortus* trickle infection (6 × 1000 L3, Group 1; 6 × 2000 L3, Group 4). Unvacinated and challenged control groups were included for both dosages (Group 3 and Group 5, respectively) and also immunostimulant control animals (Group 2) and unvaccinated and non infected lambs (Group 6). Immunization schedule with rHc23 elicited a rapid response of specific anti-rHc23 IgG (*P* < 0.05), from week 2 onwards, in the challenged lambs (Group 1) whereas no response was observed in the animals receiving the BI alone (Group 2), lambs without immunization (Group 3) or uninfected control animals (Group 6) (Fig. 1). ELISA results obtained with adult soluble extract (ASE) followed a similar pattern (not shown) consistent with the presence of Hc23 in ASE as shown in the WB (Fig. 1 insert). Comparable results were obtained with the higher dose (2000 L3/dose) (Group 4, Group 5) (not shown). Packed cell volume (PCV) values remained within the physiological range throughout the experiment (data not shown).

**Fig. 2:**
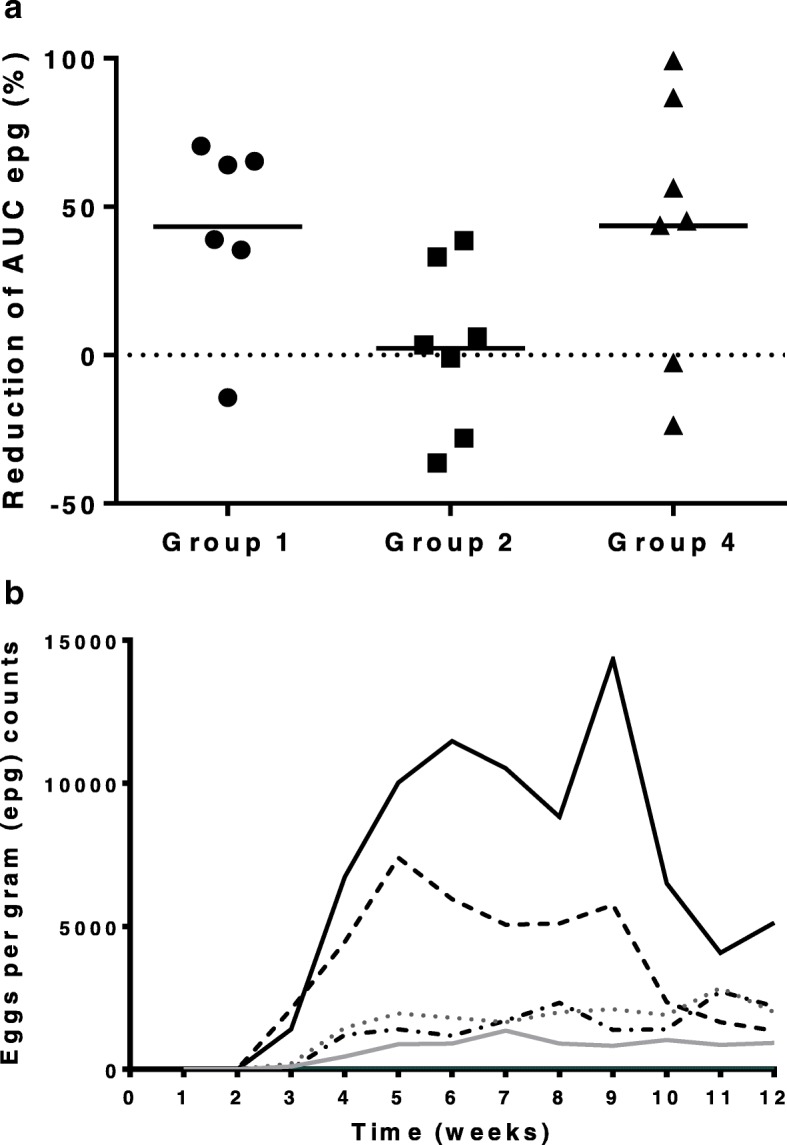
**a** Reduction (%) of individual cumulative epg values of experimental lambs after challenge with 1000 × 6 L3 (Groups 1 and 2 versus Group 3) and 2000 × 6 L3 (Group 4 versus Group 5). Group 1 and Group 4 (vaccinated with rHc23 + BI); Group 2 (non vaccinated + BI). Cumulative epg values were estimated by the trapezoidal method to determine areas under the curve (AUC). Horizontal bars represents the mean value from each group. Group 1 (●), Group 2 (■) and Group 4 (▲). **b** Median values of fecal egg counts from each experimental group along the experiment. Group 1: ^___^; Group 2: ^….^; Group 3: ^-.-.^; Group 4: ---; Group 5: ^___^

Wide variations among animals, within each group, were found in fecal egg output considering both epg pattern and total egg excretion (Fig. 2a). +Global estimation of *Haemonchus* eggs contamination of the medium along the experiment (AUC from day 21 to 84 of the experiment) showed that average AUC of vaccinated lambs exposed to the 1000 L3/dose challenge (Group 1) was reduced over 43% (AUC = 65,447.08) compared to the unvaccinated lambs (Group 3) (AUC = 115,447.5) whereas animals only receiving BI did not show any reduction (AUC = 112,787.5). Similar reductions were found in the vaccinated lambs subjected to the higher dose challenge (Group 4 AUC = 309,315) compared to the unvaccinated control group (Group 5 AUC = 547,732.5). However, differences were not statistically significant.

Pattern of egg shedding along the experiment of immunized animals was comparable with the two infective doses (Group 1 versus Group 2) (Fig. 2b). Elimination profile (median epg) of lamb groups challenged with 1000 L3/dose, both vaccinated (Group 1) and receiving the BI alone was significantly different (*P* < 0.05) to that observed in control animals (Group 3). By its part, vaccinated lambs exposed to the higher dose (Group 4) displayed a significantly lower epg from week 7th onwards when compared to the unvaccinated control group (Group 5).

Table 1 shows the *H. contortus* abomasal burden at the end of the experiment (average and median values. Helminth burdens of immunized (BI+rHc23) (Group 1 and group 4) were lower than those found in the unvaccinated lambs (Group 3 and group 5) or the lambs receiving the BI (Group 2). However, there were notable intragroup individual variations among animals and differences were not statistically significant.

**Table 1:** Experimental design of the immunization trial with rHc23 + Bacterial immunostimulant (BI) and *Haemonchus contortus* adult burden (mean ± standard error of the mean) in the abomasum of experimental lambs. Median values for total helminth counts are given in brackets *

	BI	rHc23	Challenge (L_3_)	Adult males	Adult females	Total helminths
Group 1 [6]	+	+	1000 × 6	288.5 ± 68.2	239.5 ± 43.9	528 ± 110.2 (599)
Group 2 [7]	+	o	1000 × 6	524.3 ± 98.4	481.4 ± 57.3^[1]^	1005.7 ± 150.4 (1100)
Group 3 [4]	o	o	1000 × 6	466.3 ± 118.3	486.3 ± 136	952.5 ± 253.9 (975)
Group 4 [7]	+	+	2000 × 6	578.3 ± 305.2	638.3 ± 348.2	1216.7 ± 650.8 (690)
Group 5 [4]	o	o	2000 × 6	1423.3 ± 723.4	1590 ± 792.5	3013.3 ± 1503.1 (4400)
Group 6 [4]	o	o	o	0	0	0

Trickle infection with 6 doses of 1000 L3/dose, 3 doses per week, 2 weeks: Groups 1, 2 and 3. Trickle infection with 6 doses of 2000 L3/dose, 3 doses per week, 2 weeks: Groups 4 and 5. Group 6: control group. In brackets: number of lambs from each group. +: treatment received; o: no treatment

## Discussion

Under our experimental conditions immunizations carried out with the BI + rHc23 elicited a strong antibody response against the recombinant. Reactivity of sera from immunized lambs in ELISA and WB with ASE confirms the presence of Hc23 in adult *H.contortus*. Absence of clinical signs along the experiment and lack of significance of PCV fall in non immunized and challenged lambs, besides the individual variability among lambs, was possibly due to the infective doses employed.

A decrease in the rate of establishment of incoming larvae has been described in trickle infections [9]. In our case no apparent reduction depending on the larval dosage administered (1000 L_3_ vs. 2000 L_3_) was observed at necropsy; the average value found was actually higher (> 15%) than the standard observed with this isolate in single dose infections [7, 8]. This could be related to the different duration of the infection (2 versus 15 weeks) and the variations of infection schedule.

Infection and immunization trials in this system (lambs-*H.contortus*) are hampered by individual responses [10, 11] possibly related to the genetic variability of lambs [12] and the size of the experimental groups. Actually, these differences in the individual response could reduce the significance of the findings. We have used fecal egg excretion, both epg pattern and AUC values, and abomasal adult burden at the end of experiment as estimative parameters of protection elicited by rHc23. In all cases average or median values of vaccinated animals were lower than those found in the unvaccinated control groups (Group 1 versus Group 3; Group 4 versus Group 5). However, significance of the results was variable depending on the considered parameter. Thus, epg pattern of Group 1 and group 2 was different to that observed in unvaccinated lambs (Group 3); similarly vaccinated lambs and challenged with the higher dose of *H. contortus* larvae (Group 4) were significantly different to the unvaccinated animals (Group 5) from week 7 pi onwards. Despite the lower total egg excretion found in vaccinated animals, with a reduction over 43% compared to unvaccinated and BI-stimulated lambs, no significant differences were detected in AUC; comparable results were obtained when determining the abomasal *Haemonchu*s burden at the end of the experiment. Immunomodulators, including the combination of both components of the preparation employed by us, are being increasingly used in veterinary medicine. LPS is a natural adjuvant driving, via Toll-like receptor 4 (TLR4), Th1 priming in lymphoid tissue and Th17 priming in the gut [13]. By its part, *P.acnes* has the capacity of modulating bone marrow cells [14]. Present experiment achieved lower protection levels than those obtained, with the same recombinant, using aluminum adjuvant [7, 8]. Lack of statistical significance of differences between lambs vaccinated and those treated only with the BI (Group 1 versus group 2) does not allow to exclude the possibility of BI eliciting some unspecific protection as shown in adult animals [15] despite the lower values found in rHc23-vaccinated animals. Whether or not the lower efficacy obtained is due to the infection schedule, the immunization system (adjuvant versus immunostimulant, timing) or to the size of experimental groups should be studied. Thus, the lower number of lambs in control groups (Group 3 and Group 5) and the presence of some outliers (see Fig. 2a, Group 1) precluded the detection of significant differences between experimental groups (Group 1 versus group 2) in the parameters determined. Recognition of Hc23 of *H. contortus* ASE in WB and ELISA did not show a close relation to the protection elicited by immunization (e.g. epg pattern) in the individual lambs. This suggests that protection elicited by rHc23 immunization more than probably involves both cellular and humoral components.

Partial protection against haemonchosis can be achieved using several native antigens [5, 16, 17] and vaccination using H11 from adult helminths has been initiated (www.agric.wa.gov.au/print/node/1878) and a reduced amount of protein is needed for each immunization. However the vaccination has a low performance in periparturient naïve ewes [18] and requires 5–6 revaccinations at 6 weeks interval to elicit significant levels of protection. Moreover this system uses infected and slaughtered lambs to isolate the antigen. In the absence of a culture system for adult *Haemonchus* a recombinant antigen would be an advantage on both ethical and technological grounds.

## Conclusions

Protection results obtained with different recombinants against lamb haemonchosis have been of limited value whereas rHc23 vaccination with aluminum adjuvant [7, 8] has been able to partially protect lambs subjected to single challenge. Present experiment with BI and trickle infections in animals yielded suggestive although inconclusive results. More experimental work is needed with rHc23, particularly on refinement of dose, adjuvants, immunization schedules, and involving higher number of animals. However, taken together the results obtained previously and these from the present experiment with BI, point towards the interest of rHc23 as a promising candidate to develop a recombinant vaccine for lamb haemonchosis [19].

## Methods

### Parasites and immunizing material

The *H.contortus* isolate was originally supplied by Merck, Sharp & Dohme (Madrid, Spain) and has been maintained in our facilities for over 30 years by serial passage in lambs. Infective larvae (L3) were obtained by faecal culture, recovered using the Baermann technique, cleaned by partial purification on filter paper and stored at 4 °C in tap water until use [20]. Recombinant protein rHc23 was obtained as previously described [7, 8]. For immunization trial, the protein was resuspended in PBS and protein concentration was determined by RC/DC protein assay (Bio Rad).

### Lambs and experimental design

Female 5–6 months old Entrefino type lambs were obtained from local producers (“Antón Codesal, Sociedad Cooperativa”, Cerezal de Aliste, Zamora, and “El Navajo”, Mondéjar, Guadalajara, Spain). Lambs were subjected to quarantine and housed under helminth-free conditions at the facilities of the Faculty of Veterinary Medicine, UCM (ES280790000137), fed commercial pellets (Rubio Sanidad y Alimentación Animal, Madrid, Spain), hay and tap water ad libitum*.* Lambs (32) were divided in a stratified manner (live weight) onto six groups. Group 1 (6 lambs) and Group 4 (7 lambs) were immunized with 100 μg/dose rHc23 + bacterial immunostimulant (LPS of *Escherichia coli* + *Propionibacterium* extract) (Lab. Calier, Barcelona) (3 mL) on days − 2, 0, 7 and 14 of the experiment by intramuscular injection in the legs. Group 2 (7 lambs) was the immunostimulant control group and only received the BI on the same days. Group 3 (4 lambs) and Group 5 (4 lambs) did not receive any immunization or bacterial immunostimulant. On days 0, 2, 4, 7, 9 and 11 of the experiment lambs were challenged with 1000 L_3_ of *H. contortus* (Group 1, Group 2 and Group 3) or 2000 L3 (Group 4, Group 5). Group 6 (4 lambs) remained as the non immunized and non challenged group (Table 1). Animals were daily observed for adverse reactions or clinical signs of the infection. Bacterial immunostimulant was administered following the manufacturer’s indications. At the end of the experiment lambs were slaughtered by stunning and throat slitting, as regulated by current legislation (EC 1099/2009; Royal Decree 53/2013), at a local abattoir (Villarejo de Salvanés, Madrid).

### Clinical, immunological and parasitological follow up

Individual fecal samples were taken from the rectum once a week until the end of the experiment. Fecal egg counts (eggs per gram, epg) were determined using a modified McMaster technique [20]. Cumulative epg counts were calculated using the trapezoidal method to determine areas under the curve (AUC) of animals and groups. Blood was collected weekly by jugular venipuncture using evacuated tubes with anti-coagulant to determine PCV and without anti-coagulant to obtain sera. After slaughter of the lambs their abomasums were removed to determine helminth burdens [21].

Adult *H.contortus* soluble extract (ASE) was obtained by freezing and thawing cycles and protein concentration was determined with RC/DC protein assay (Bio Rad). Peripheral antibody response was determined by ELISA using microtiter 96-well plates (Nunc, Denmark) coated with 5 μg/mL ASE or 1 μg/mL rHc23 [22]. Sera were used at 1/200 dilution (1 h at 37 °C) and the conjugate (alkaline phosphatase-labelled rabbit anti-sheep IgG, Sigma–Aldrich, USA) was used at 1/8000 (ASE) and 1/32000 (rHc23) dilution (1 h at 37 °C). Color was developed with 1 mg/mL 4-nitrophenil phosphate disodium salt hexahydrate (Sigma–Aldrich, USA) for 30 min at 37 °C and optical density (OD) was measured with Multiskan GO microplate reader (Thermo Fisher Scientific, USA) at 405 nm. *H.contortus* ASE was fractionated with 12.5% SDS-PAGE and Western blot was performed as described [22]. The conjugate was horseradish peroxidase (HRP)-labeled donkey anti-sheep IgG (Sigma–Aldrich, USA) diluted 1/1000 (1 h, 37 °C). Color was developed with 4-chloro-1-naphtol (0.5 mg/mL) and the reaction stopped with distilled water. Molecular weight (MW) markers were from GE Healthcare (UK).

### Statistical analysis

Pattern of fecal egg counts along the experiment were analyzed using a generalized linear model considering a negative binomial distribution with a logarithmic link function and a symmetric correlation structure. Cumulative fecal egg output was estimated using the trapezoidal method to determine areas under the curve (AUC) of the animals and groups from day 21 to 84 of the experiment. Values of AUC and helminth burdens (males, females, total helminths) among groups were compared using a negative binomial regression. Group 1 and Group 2 were compared to the unvaccinated control animals receiving the same challenge (Group 3). Vaccinated and challenged animals with the higher dose (2000 L3/dose) (Group 4) were compared to the unvaccinated animal group (Group 5). Statistical analysis was performed using Stata 15 software and for all tests, *P <* 0.05 was set as level of significance. Figures were done with GraphPad Prism Version 6.0 software.

## Data Availability

Data will be available directly from the authors if required (mcayensa@ucm.es; jmalunda@ucm.es).

## Abbreviations

ANOVA: Analysis of Variance
ASE: Adult Soluble Extract
AUC: Area Under the Curve
BI: Bacterial Immunostimulant (LPS of *Escherichia coli* + *Propionibacterium acnes* extract)
e.g.: *exempli gratia*
ELISA: Enzyme Linked Immuno Sorbent Assay
epg: eggs per gram
HRP: Horse Radix Peroxidase
L_3_: third stage larvae
LPS: lipopolysacharide
OD: Optical Density
PCV: Packed Cell Volume
rHc23: Recombinant of *Haemonchus contortus* of molecular mass 23 KDa
SDS-PAGE: Sodium Dodecyl Sulphate – Polyacrylamide Gel Electrophoresis
WB: Western blot
